# Nuclear blebs are rupture‑prone plastic deformations

**DOI:** 10.1080/19491034.2026.2735710

**Published:** 2026-11-04

**Authors:** Mindy Zheng, Andy Li, Andrew D. Stephens

**Affiliations:** a Biology Department, University of Massachusetts Amherst, Amherst, MA, USA; b Molecular and Cellular Biology, University of Massachusetts Amherst, Amherst, MA, USA

**Keywords:** Micromanipulation, NLS-GFP, nuclear bleb, nuclear rupture, plasticity

## Abstract

Abnormal nuclear morphology is a hallmark of various human diseases. Nuclear blebs are herniations of the nucleus found in many diseased nuclei and often cause nuclear rupture. While nuclear blebbing and rupture are known to cause dysfunction, the relationship between nuclear blebbing and rupture, longevity, and plasticity remain unclear. We performed time-lapse imaging of nuclear localization signal green fluorescent protein (NLS–GFP) to track the history of individual blebbing events. Nuclear blebs typically form within 12 hours after the cell exits mitosis. We find that nuclear blebs predominantly form first, leading to multiple nuclear ruptures. Nuclear blebs are either long-lived, with many requiring entry into mitosis for reabsorption, or short-lived largely due to being the second bleb on a nucleus. Nuclear blebs remain upon both in-cell actin depolymerization and nuclear isolation through micromanipulation. We reveal that nuclear blebs are plastic deformations that provide a persistent site of nuclear rupture.

## Introduction

Nuclear blebs fall under the broad classification of abnormal nuclear morphology, a hallmark of many human diseases. Nuclear blebs are distinct protrusions of the nucleus greater than 1 µm in length from the main nuclear body that are commonly associated with nuclear rupture [1,2]. Nuclear blebs are a distinct herniation of the nucleus that differs from nuclear lobulations, which are a less distinct, more broad-scale alteration of nuclear shape that are not associated with nuclear rupture, as occurs upon loss of lamin A/C [2–5]. Nuclear rupture is the rapid loss of nuclear compartmentalization and is well reported to cause nuclear dysfunction [6]. This improper mixing of nuclear and cytoplasmic components disrupts different cellular processes, including DNA damage, inflammation, transcription, and cell cycle control [2,7–13]. While it is known that nuclear blebs are associated with nuclear rupture, it is unclear whether the bleb appears before rupture or whether rupture occurs before the bleb forms.

Repair of the nuclear membrane from nuclear rupture generally occurs within 10–15 min after rupture. Nuclear blebs persist even after the nuclear membrane has been repaired post-nuclear rupture [1,9,10,14]. Despite the nuclear bleb frequently lingering post-rupture, normal nuclear morphology can be restored. We previously reported that nuclear bleb stability is partially dependent on transcription activity, as disruption of transcription activity results in increased rates of nuclear bleb reabsorption, returning the nucleus to a normal elliptical shape [15,16]. However, under normal conditions it remains unclear how long nuclear blebs will persist. We hypothesize that many nuclear blebs persist until mitosis. This hypothesis is supported by a qualitative connection between the persistence of nuclear blebs until mitosis [17] and the loss of nuclear blebs upon mitosis in colorectal adenocarcinoma cells [18].

Nuclear blebs could be an elastic deformation that requires constant force to maintain it or a plastic deformation that requires no external force for maintenance but an active force to reabsorb it. Whole-nucleus deformation during force measurements shows that the nucleus is an elastic element [19–22]. However, smaller deformations like nuclear blebs have not been investigated. If the nuclear bleb can rebound back to its original morphology after the removal of force, then it is considered an elastic deformation. Alternatively, if the nuclear morphology is not restored to its normal shape after removal of external force, it is considered a plastic deformation [23]. Work by others has shown that abnormal nuclear shape behaves plastically as it remains upon cutting actin filaments [24], as actin confinement is essential to nuclear blebbing [2,14,25].

We used time-lapse imaging of nuclear localization signal (NLS)-GFP in wild‑type (WT) mouse embryonic fibroblasts (MEF) and perturbations that increase nuclear blebbing to follow the life history of a nuclear bleb. We tracked nuclear bleb formation, nuclear rupture, reabsorption, mitosis, and elastic vs. plastic behaviors. Our findings provide insight into the biophysical principles underlying nuclear blebs.

## Results

### Nuclear blebs form before nuclear rupture.

Many previous works have reported that nuclear blebbing and rupture are correlated, but the order remains unclear. To track nuclear blebbing formation relative to rupture, we performed time-lapse imaging of MEFs with NLS-GFP. We tracked the nuclear bleb formation events in WT MEF cells and in two perturbations that increase nuclear blebbing through treatment with valproic acid (VPA), a histone deacetylase inhibitor, and lamin B1 knockout (*Lmnb1-/-*). Static nuclear blebbing was measured at 1.8 ± 0.5% in WT, which increases to 10.7 ± 0.7% in VPA and 10.3 ± 0.5% in *Lmnb1-/-* (biological triplicates, *n* > 300). While increased nuclear blebbing and rupture frequency have been reported multiple times for WT cells compared to VPA-treated or *Lmnb1-/-* cells [1,2,15,16], the ordering remains unclear.

We categorized the observed nuclear blebs into two different formation events: ‘Bleb→Rupture’ and ‘Rupture→Bleb’ (Figure 1A). Bleb→Rupture indicates a nuclear bleb forming before nuclear rupture. Rupture→Bleb indicates a nuclear bleb forming shortly after rupture. For only determining the order of nuclear blebbing and rupture, we excluded 17% of nuclear blebs which showed unresolvable bleb formation and rupture in which they occurred simultaneously while 10% of blebs showed no rupture. We observed that across all cell conditions, over 80% of nuclear blebs formed first, then rupture followed (Figure 1B). Conversely, only 20% of nuclear blebs formed after a nuclear rupture. Analysis in HT1080 nuclear blebs recapitulates > 80% of resolvable events the nuclear bleb forms before rupture (Sup. Figure 1B). Thus, these data reveal that nuclear blebs predominantly form before nuclear rupture.

**Figure 1.**
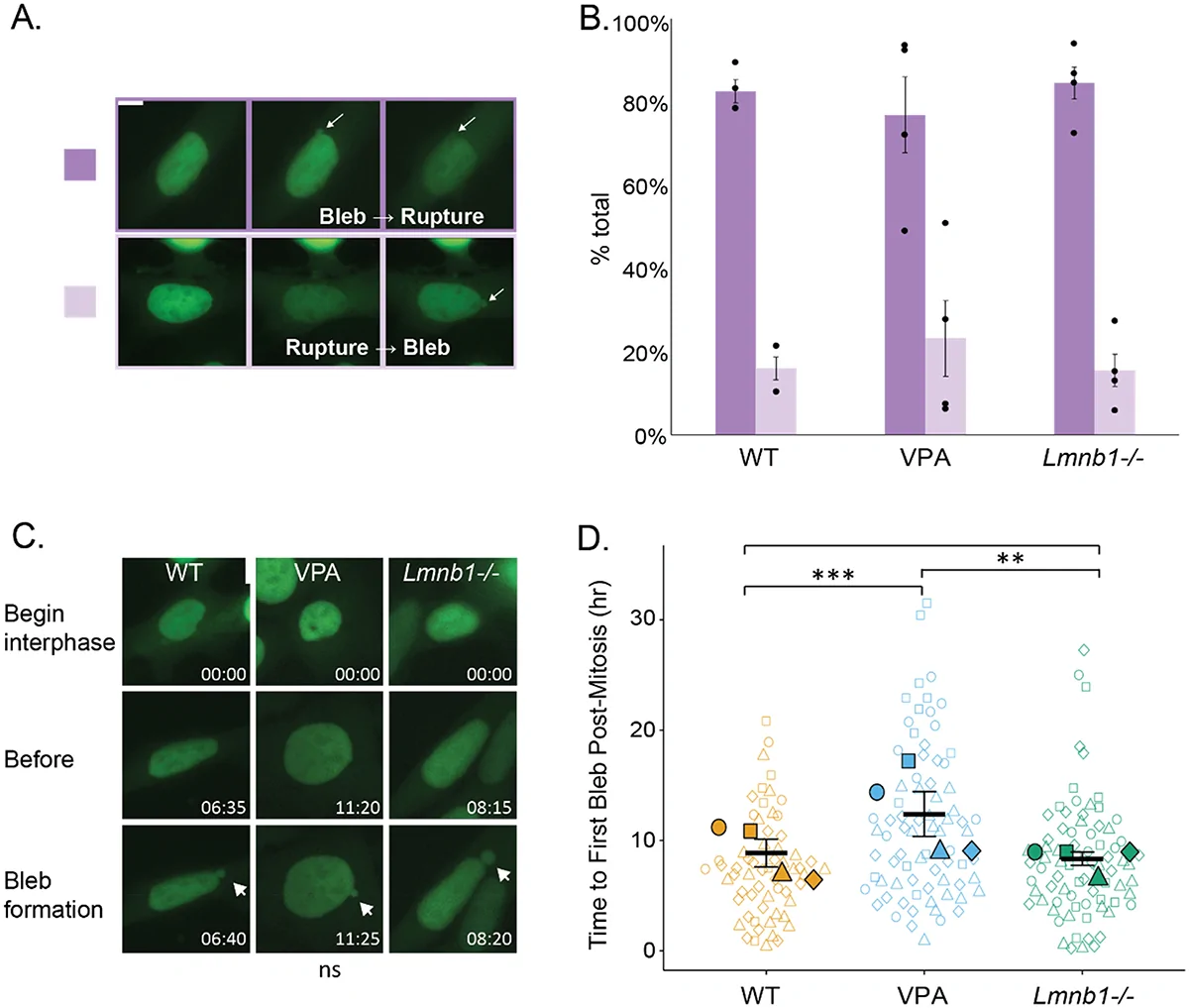
Time-lapse imaging with NLS-GFP reveals that nuclear blebs form before nuclear rupture. (A) Example images of MEF WT nuclei with NLS-GFP and (B) a graph of nuclear blebbing formation percentages: Bleb→Rupture and Rupture→Bleb in MEF cell conditions, wt, chromatin decompaction (VPA), and *Lmnb1-/-*. (C) Example images of bleb formation over a 36-h time‑lapse in NLS-GFP with timestamps (hr:min) and (D) superplots of the time points at which a nuclear bleb forms post-mitosis in wt, VPA, and *Lmnb1-/-*. Four biological replicates for all data, >15 cells per replicate. Error bars represent the standard error, and statistical tests are one-way ANOVA with a post-hoc Tukey test; significance is denoted by * = *p* < 0.05, ** = *p* < 0.01, and *** = *p* < 0.001. A white arrow indicates a nuclear bleb. Scale bar = 10 μm.

To determine when nuclear blebs formed, we tracked the time points at which nuclear blebbing occurred post-mitosis nuclear reformation (Figure 1C). Nuclear blebs form from normally shaped nuclei during interphase. We observed that nuclear blebs typically formed within 12 h after exiting mitosis, in agreement with our previous work showing that nuclear blebs form mostly in G1 [17]. VPA-treated cells had blebs that formed slightly later than WT cells, whereas bleb formation in *Lmnb1-/-* cells did not differ greatly from WT (Figure 1D). Overall, the data suggest that nuclear blebs predominantly form earlier in the cell cycle and the nuclear rupture follows.

### Repeated nuclear rupture follows shortly after nuclear bleb formation

After demonstrating that nuclear blebs lead to nuclear rupture in the majority of cases, we hypothesize that nuclear rupture occurs soon after a nuclear bleb has formed. To test our hypothesis, we recorded the time between when the bleb forms and its first nuclear rupture (Figure 2B). We find that over 70% of nuclear ruptures occur within 25 min of nuclear bleb formation (Figure 2B). Thus, the data show that nuclear rupture follows soon after the formation of a nuclear bleb.

**Figure 2.**
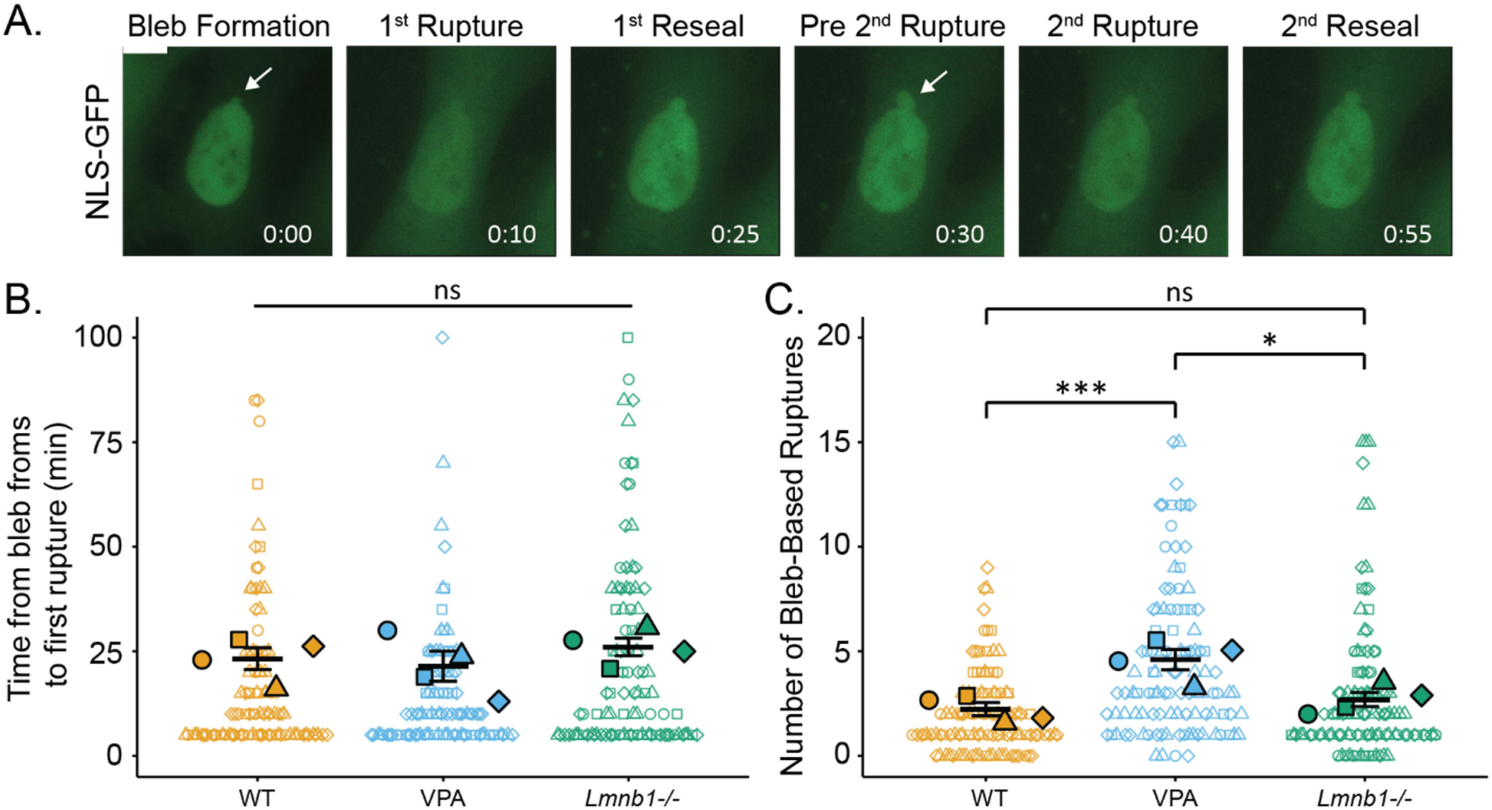
Time-lapse imaging reveals that blebbed nuclei rupture more frequently. (A) Example images of time-lapse imaging of MEF wt nuclei with NLS-GFP showing multiple nuclear ruptures, all occurring in the same nuclear bleb. Time stamp is hr:min. (B) Superplots of the time from bleb formation to the first nuclear rupture in wt, VPA, and *Lmnb1-/-*. (C) Superplots of the number of nuclear ruptures per nucleus in MEF cell conditions, wt, VPA, and MEF *Lmnb1-/-*. Four biological replicates for all data, >15 cells per replicate. Error bars represent the standard error, and statistical tests are one-way ANOVA with a post-hoc Tukey test; significance is denoted by * = *p* < 0.05, ** = *p* < 0.01, and *** = *p* < 0.001. A white arrow indicates a nuclear bleb. Scale bar = 10 μm.

To determine whether nuclear blebs result in repeated ruptures, we counted the number of ruptures per nuclear bleb over its entire lifetime (Figure 2A). We found that more than one rupture occurred per nuclear bleb across cell conditions (Figure 2C). WT nuclear blebs resulted in 2.2 ± 0.3 ruptures per nuclear bleb. The number of ruptures per nuclear bleb in *Lmnb1-/-* cells did not differ greatly from WT cells. VPA-treated cells had nuclear blebs with a significantly increased rupture frequency of 4.6 ± 0.5 ruptures per nuclear bleb. Thus, nuclear blebs lead to repeated nuclear ruptures.

### Nuclear blebs are long-lived

While nuclear blebs have been reported for almost 20 years, the longevity of nuclear blebs remains unclear. We hypothesized that nuclear blebs, which form during interphase, could exist until mitotic nuclear breakdown, or blebs could be reabsorbed during interphase. We tracked nuclear blebs from their formation to their disappearance. We specified the disappearance of nuclear blebs into two categories: ‘Reached Mitosis’ and ‘Reabsorbed.’ Nuclear blebs that reach mitosis are blebs that form and stay until the nucleus undergoes mitosis (Figure 3A). Reabsorbed blebs are nuclear blebs that disappear after formation but before the nucleus undergoes mitosis (Figure 3B). Nuclear blebs were considered absent when the bleb did not protrude more than 1 µm in length from the nuclear body. Overall, the number of nuclear blebs that reached mitosis or were reabsorbed was split 50/50, suggesting underlying complexities.

**Figure 3.**
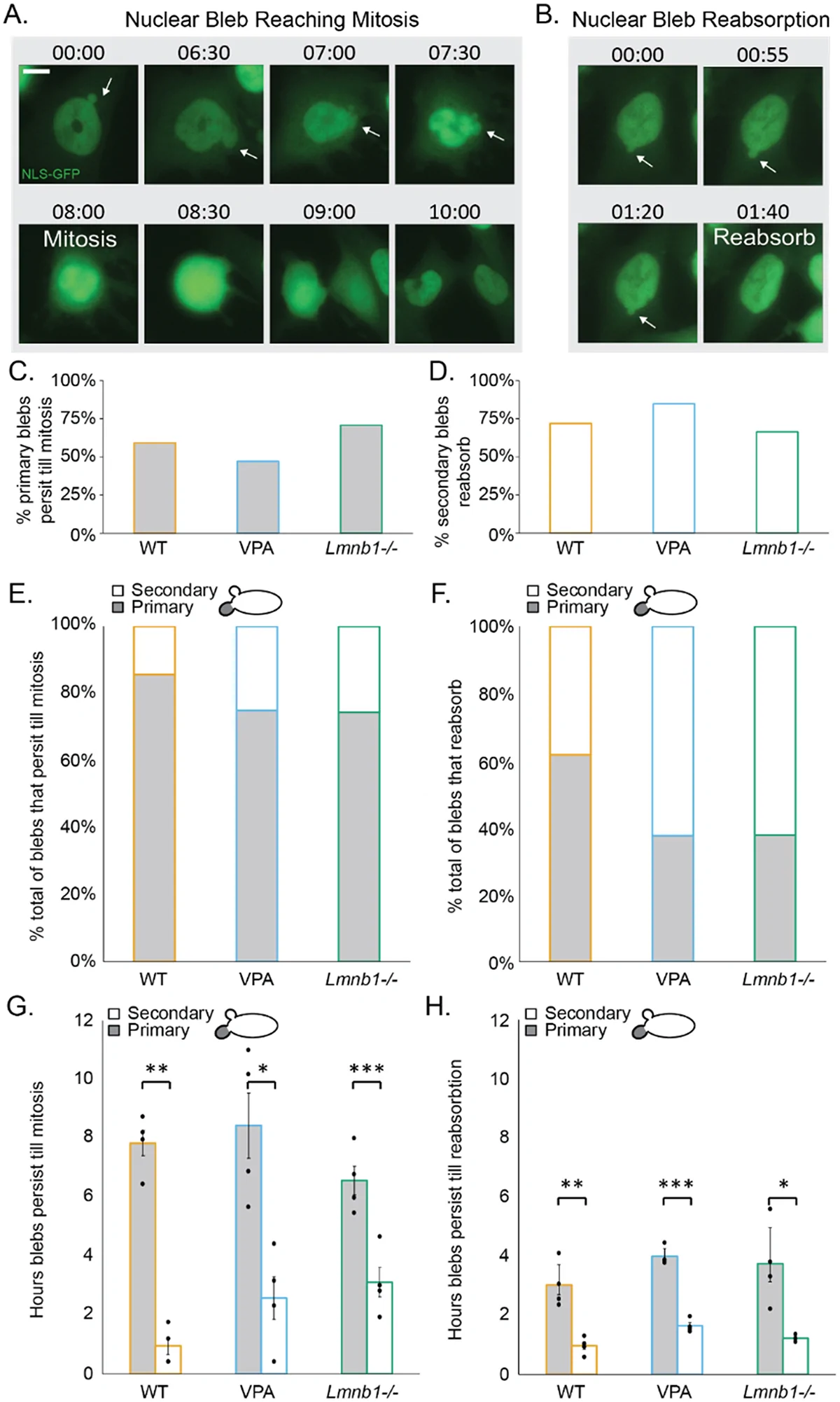
Time-lapse imaging shows that nuclear blebs persist for hours until mitosis or reabsorption. (A) Example images of a MEF WT nuclear bleb with NLS-GFP reaching mitosis with timestamps and (B) a MEF wt nuclear bleb that gets reabsorbed with timestamps. Graph of percentages of (C) primary (gray) nuclear blebs that reach mitosis vs. (D) secondary (white) nuclear blebs that are reabsorbed in MEF cell conditions wt, VPA, and *Lmnb1-/-*. Graph of percentages of primary (gray) vs. secondary (white) nuclear blebs that (E) reach mitosis or (F) are reabsorbed in MEF cell conditions wt, VPA, and *Lmnb1-/-*. Bar graphs of the times a nuclear bleb lasts (G) until mitosis or (H) reabsorption for primary (gray) vs. secondary (white) nuclear blebs in MEF cell conditions wt, VPA, and *Lmnb1-/-*. Four biological replicates for all data, >15 cells per replicate. Error bars represent the standard error, and statistical tests are two-tailed paired students’ t-test; significance is denoted by * = *p* < 0.05, ** = *p* < 0.01, and *** = *p* < 0.001. A white arrow indicates a nuclear bleb. Scale bar = 10 μm.

To determine whether this near‑equal split was due to bimodal behavior, we investigated the differences between blebs that persist until mitosis and those that reabsorb during interphase. Specifically, we tracked primary blebs, classified as the only bleb on a nucleus or the longest-lived bleb on a nucleus with multiple blebs. We also tracked secondary blebs, which we classified as the 2^nd^ or 3^rd^ bleb on the nucleus. Primary blebs reach mitosis > 54% of the time, while secondary blebs > 75% are reabsorbed in interphase (Figure 3, C and D). While these behaviors are drastic, we aimed to further determine whether primary and secondary blebs underlie bimodal behavior.

To better understand the differences between primary and secondary blebs, we analyzed their percentage and hours of bleb persistence in both blebs that reached mitosis and those that reabsorbed in interphase. Primary nuclear blebs make up the large majority of the nuclear blebs that reached mitosis (>75%, Figure 3C). Even when only considering blebs that reach mitosis, primary blebs persist for a significantly longer time—between 6.5–8.5 h compared to secondary blebs, which last 1–3 h on average (Figure 3D). As expected, nuclear blebs that reabsorbed in interphase lived for a significantly shorter time than blebs that lasted until mitosis. For nuclear blebs that reabsorbed, secondary blebs were the majority in VPA and *Lmnb1-/-* at > 60% (Figure 3E). Nuclear blebs that are reabsorbed still show a significant difference between primary blebs that last 3–4 h and secondary blebs that last 1–1.5  h on average (Figure 3F). Thus, the bimodal behavior of nuclear blebs in MEFs is due to long-lived primary blebs that majority last until mitosis and short-lived secondary blebs that majority reabsorb in interphase.

To determine whether nuclear bleb lifetime was similar in other cell lines, we tracked HT1080 blebs. Overall, HT1080 nuclei presented even more long-lived nuclear blebs, independent of whether the bleb was the first, second, or third on the same nucleus. Nuclear blebs persisted until mitotic entry in >75% nuclei for both WT and VPA-treatment (Sup. Figure 1, A and C). Overall nuclear bleb lifetime was 13.1 ± 1.3 hours in WT and 9.5 ± 0.7 hours in VPA-treated (Sup. Figure 1D). Interestingly, for HT1080 nuclei, primary and secondary blebs behaved similarly overall. Thus, nuclear blebs across cell lines display long-lived persistence until mitotic entry with variability in the persistence of the secondary blebs formed on the same nucleus.

### Nuclear blebs display plasticity

Nuclear blebs are abnormal deformations of the nucleus that could be elastic and rebound upon removal of cellular forces, or could be plastic deformations that remain. Actin contraction and compression is reported to be necessary for nuclear bleb formation [2,14,25–27]. However, it remains unknown if actin is necessary to maintain already formed nuclear blebs. To determine if nuclear blebs are elastic or plastic, we tracked blebbed nuclei upon depolymerization of actin via latrunculin A for 1-hour post-treatment (Figure 4A). Nuclear blebs remained upon removal of actin compression, though the nuclear body and bleb did round up. Thus, in-cell data show that nuclear blebs remain plastically deformed upon depolymerization of actin.

**Figure 4.**
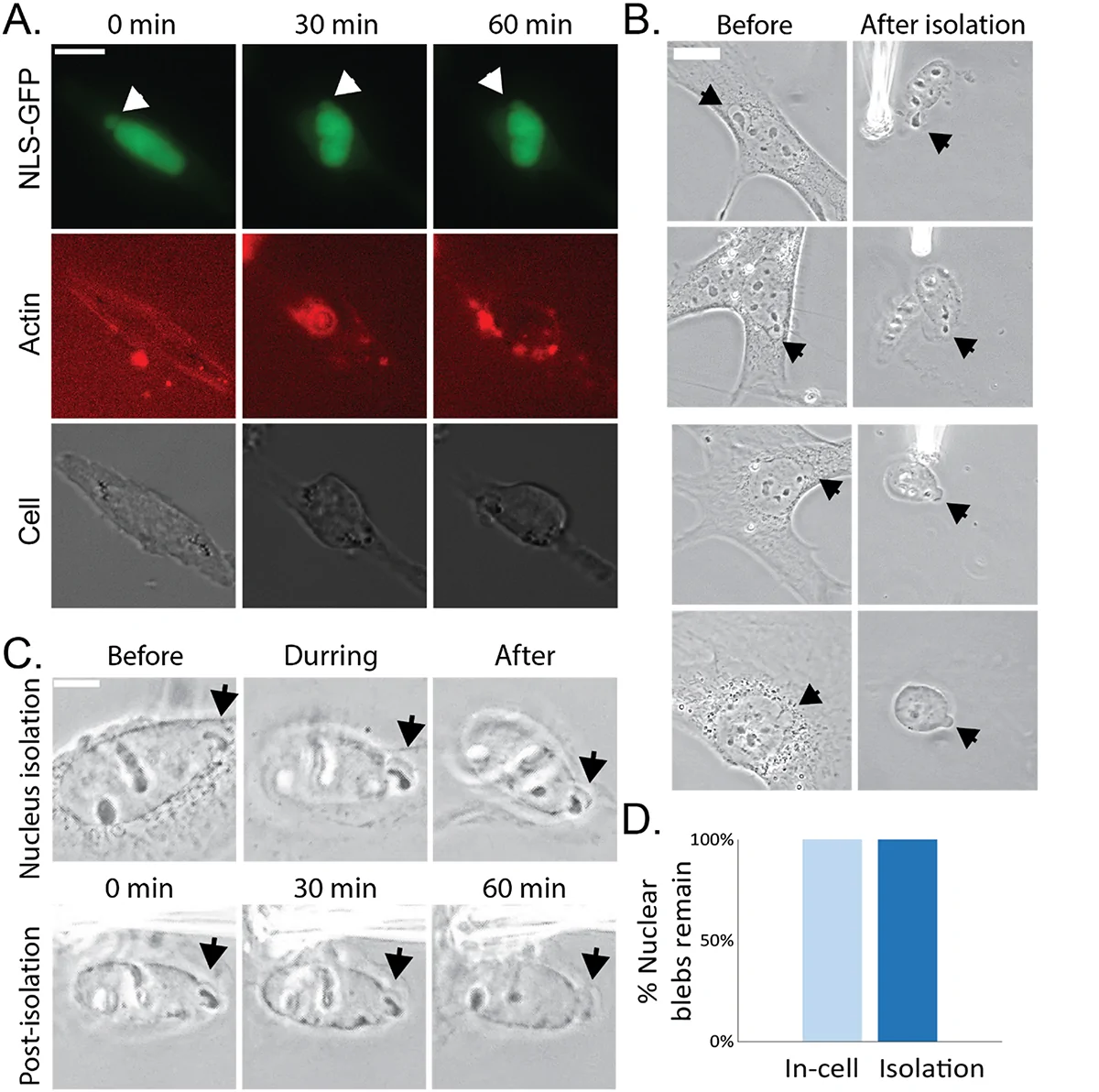
Nuclear blebs persist after actin depolymerization and nuclear isolation. (A) Example images of the nuclear bleb and body with NLS-GFP, actin stained with SPY-Actin, and the cell with transmitted light over the course of 1 h after actin depolymerization with latrunculin A (LatA) treatment. (B) Example images of nuclear blebs in a live cell before and after nucleus isolation using micromanipulation. (C) Time course of nucleus isolation and 1 h after. (D) Graph of the percentage of blebs that remained in‑cell (*n* = 6) and in isolation (*n* = 20). Arrows indicate a nuclear bleb. Scale bar = 10 μm.

Lifetime dynamics in Figure 3 suggest that primary and secondary nuclear blebs may be physically different. Thus, we conducted actin depolymerization on nuclei with two blebs. Both nuclear blebs remained after an hour or more following actin depolymerization in all measured events (*n* = 4 nuclei with two nuclear blebs, Sup. Figure 2A). Thus, both primary and secondary nuclear blebs remain plastically deformed. Furthermore, we tested primary vs. secondary nuclear bleb chromatin composition. Nuclear bleb-to-body ratios can be used to determine bleb composition while normalizing for different stains and immunofluorescence antibodies [28–30]. Measurements show that primary and secondary nuclear bleb chromatin composition is the same for DNA, H3, and the constitutive heterochromatin marker H3K9me3 in MEF and HT1080 cells (Sup. Figure 2B). Thus, primary and secondary nuclear blebs display similar plasticity upon actin depolymerization and have similar chromatin composition.

An alternative approach to remove the nucleus from cellular forces is to use micromanipulation to isolate the nucleus from the cell (Figure 4B). In this technique, micropipettes spray a gentle detergent to break open the cell membrane and isolate the nucleus away from the cell. We used MEF vimentin null cells that are amenable to nuclear isolation by micromanipulation from living cells without using actin depolymerization [31]. For every blebbed nucleus isolated by micromanipulation, the bleb remains present, suggesting that it is a plastic deformation. Tracking for 1 h shows no change or sign of reabsorption as the bleb remains (Figure 4C). This data provides a recapitulation of the in-cell actin depolymerization data. For both in-cell actin depolymerization and nuclear isolation, 100% of nuclear blebs imaged remained intact (Figure 4D). Thus, both in-cell and isolation data reveal that nuclear blebs are plastic deformations of the cell nucleus.

## Discussion

### Nuclear bleb formation precedes nuclear rupture.

Previous studies have established the relationship between nuclear blebbing and nuclear rupture, but the order in which they occur has yet to be extensively quantified. Our data report that nuclear rupture is a result of nuclear blebbing, rather than the other way around (Figure 1B). Interestingly, a small portion (10%) of nulcear blebs did not result in rupture. The existence of nuclear blebs that form without rupture strongly supports that overall nuclear blebs do not require a preceding rupture to form. We find that this sequence of events remains consistent across MEF cell conditions, suggesting that it is a shared characteristic of nuclear blebs.

We again show that blebs form early in the cell cycle (Figure 2), and that blebs that form in G1 persist into G2, with many persisting until nuclear envelope breakdown at mitosis [17]. With nuclear bleb formation being an early interphase phenomenon, nuclear blebs may linger around for longer as the nucleus moves through the cell cycle. While nuclear rupture and repair is relatively quick—on the order of 10 min [2,32], nuclear blebs are much longer lived, lasting on the order of hours. In particular, the majority of nuclear blebs persist until mitosis (Figure 3), suggesting that a full breakdown and reset of nuclear structure is required to restore normal nuclear shape. The longevity of a nuclear bleb has implications for cellular dysfunction, either through multiple nuclear ruptures [6] or independently of ruptures [29,33]. Although the mechanism of how a nuclear bleb forms remains unknown, our novel data reveal that nuclear blebbing occurs independently of nuclear rupture.

### The cell nucleus is elastic, but nuclear blebs are plastic.

The elastic and plastic properties of the nucleus are scale dependent. Whole‑nucleus mechanics measured by micromanipulation dual‑micropipette force‑extension measurements show that the whole nucleus behaves elastically as the nucleus is extended and then returned to its original state. The nucleus maintains this elastic force response for 3–6 pulls [21]. Similarly, light sheet imaging of atomic force microscopy indentations shows elastic rebound [22]. Repeated measurements of the same nucleus provide similar force-deformation responses, suggesting that mechanical components remain intact. Thus, extension and compression force measurements report that the nucleus is an elastic material.

Oppositely, nuclear blebs show no elastic rebound (Figure 4). Upon actin depolymerization or nucleus isolation, nuclear blebs remain. Thus, this local deformation behaves as a plastic deformation, which is different from whole nuclear elasticity. Furthermore, our data agree with previous work showing that abnormal nuclear shape remains upon cutting of the cytoskeleton in breast cancer model cells [24]. While micropipette aspiration force measurements applied to the whole nucleus result in elastic rebound [19,20], aspiration of a portion of the nucleus into the micropipette, causing greater than 200% local deformation, causes plastic deformation of the nucleus [34,35]. This scale is similar to nuclear blebs that range between 1 and 5 µm of local herniation. Even whole nuclear deformations can become plastic after successive migration through constrictions [11,36].

Plasticity of the nuclear bleb suggests that major mechanical components break. In fact, data support the idea that both major mechanical components of chromatin and lamins break upon bleb formation. DNA and histones are both shown to decrease in density in the nuclear bleb relative to the body [28,30,37]. Lamin A/C is shown to be absent from newly forming blebs [9] and is only re-recruited after nuclear envelope rupture [29,38]. This plasticity is relevant to human disease because it suggests that blebs passively remain and thus can continue to cause cellular dysfunction.

If nuclear blebs are plastic deformations, then how do a significant portion of nuclear blebs lead to reabsorption back to normally shaped nuclei? Our data reveal that primary blebs are long lived while secondary blebs tend to reabsorb, suggesting different underlying physical behaviors (Figure 3). However, our data also reveal that primary and secondary nuclear blebs both remain plastically deformed upon actin depolymerization and have similar chromatin composition (Sup. Figure 2A). An alternative hypothesis to explain differences in primary vs. secondary nuclear bleb lifetime is that active cell forces are involved. Plastic deformation does not rule out active forces that can recompact the chromatin and/or lamins to reabsorb a nuclear bleb. In fact, past reports show that nuclear bleb reabsorption is due to chromatin compaction [39]. Our work also shows that transcriptional activity through chromatin motion is essential to nuclear bleb stability. Upon inhibition of transcriptional activity, nuclear blebs can form but reabsorb immediately after their first rupture [15]. Furthermore, reactivation of transcriptional activity quickly causes nuclear blebs to form and stabilize into plastic deformations [16]. We recently showed that chromatin stiffening and transcriptional deactivation via topoisomerase 1 inhibition could cause nuclear bleb reabsorption in 1 to 4 hours [40]. Finally, no data report that active chromatin remodelers SWI/SNF BRG1 or cohesin loop extrusion are essential to nuclear blebbing. Oppositely, loss of BRG1 causes nuclear blebbing and increased size due to changes to higher order chromatin [41,42] while loss of cohesin causes multilobulated nuclei due to mitotic failure [43]. Further research will be required to understand active processes that can reabsorb nuclear blebs to maintain nuclear shape and function relevant to human health and disease.

## Materials and methods

### Cell culture

MEFs were previously described [44–46]. MEF WT and lamin B1 knockout (*Lmnb1-/-*) cells were cultured in DMEM (Corning) supplemented with 10% fetal bovine serum (FBS; HyClone) and 1% penicillin/streptomycin (P/S; Corning). Cells were incubated at 37 °C in 5% CO_2_ and passaged at around 80% confluency every 2 to 3 days.

### Drug treatments

MEF WT cells were first plated in DMEM complete and incubated for 24 h before being treated with 4 mM valproic acid (VPA, 1069–66-5, Sigma [47]). VPA was dissolved in cell media 24 h before time-lapse imaging.

### Time‑lapse imaging and analysis

As previously described, we used established MEF NLS–GFP stable cell lines to quantify nuclear shape and rupture [2,15]. Live-cell images were acquired with Nikon Elements software on a Nikon Instruments Ti2-E microscope, Orca Fusion Gen III camera, Lumencor Aura III light engine, TMC CleanBench air table, and a 40× air objective (N.A. 0.95, W.D. 0.66, MRH00401). Live-cell time-lapse imaging was possible using the Nikon Perfect Focus System and Okolab heat at 37 °C, with supplemental humidity and a 5% CO_2_ stage top incubator (H301).

Cells were imaged in 4-well cover glass dishes. Time-lapse images were captured at 5-min intervals over 36 h. They were analyzed by tracking nuclear bleb formations and ruptures, reabsorption events, and mitosis over the duration of the movie. Timepoints of when these events occurred were recorded. As outlined previously [17], the images were analyzed in NIS-Elements.

*Lmnb1-/-* cells treated with latrunculin A (LatA, Cayman Chemical;10 µM) were imaged in 4-well cover glass dishes. Time-lapse images were captured at 5-min intervals over 2 h. The body and bleb of each nucleus were measured by drawing regions of interest (ROIs). Measurements of nuclear area were recorded and exported to Microsoft Excel.

### Micromanipulation

MEF Vimentin null (MEF *V-/-*) cells were grown in a 28 mm micromanipulation well. Use of MEF *V-/-* cells allows isolation of nuclei without the use of actin depolymerization. Micropipettes were fashioned using a Flaming/Brown micropipette puller p-97 (Sutter Instruments) with thin wall glass capillaries from World Precision Instruments TW100-6. Pre- and post-isolation images of the nucleus were taken while recording. Nuclei were isolated from living cells with a spray micropipette filled with Triton X-100 (0.05%) in PBS. This isolation was conducted first by spraying each pole of the nucleus over a span of 10 s. Mechanical isolation via a second pipette containing PBS was performed to move the newly isolated nucleus away from the cell body; slight aspiration of the nucleus into the pipette to anchor it in place while the nucleus remained suspended in cell media for up to 1 h. A total of 20 blebbed nuclei were isolated, and 1-h tracking of bleb persistence was completed for six nuclei.

## Supplementary Material

Supplemental Figures 1 and 2.pdf

## Data Availability

All raw data that is available https://doi.org/10.6084/m9.figshare.32513595. Image dataset can be made available upon request.

## References

[1] Vargas JD, Hatch EM, Anderson DJ, et al. Transient nuclear envelope rupturing during interphase in human cancer cells. Nucleus. 2012;3(1):88–11. doi: 10.4161/nucl.18954 PMC3342953 22567193

[2] Pho M, Berrada Y, Gunda A, et al. Actin contraction controls nuclear blebbing and rupture independent of actin confinement. Mol Biol Cell. Vol 35, No. 2. 2023: mbcE23070292. 10.1091/mbc.E23-07-0292 PMC10881147 38088876

[3] Lammerding J, Schulze PC, Takahashi T, et al. Lamin A/C deficiency causes defective nuclear mechanics and mechanotransduction. J Clin Invest. 2004;113(3):370–378. doi: 10.1172/JCI200419670 PMC324542 14755334

[4] Chen NY, Kim P, Weston TA, et al. Fibroblasts lacking nuclear lamins do not have nuclear blebs or protrusions but nevertheless have frequent nuclear membrane ruptures. Proc Natl Acad Sci USA. 2018;115(40):10100–10105. doi: 10.1073/pnas.1812622115 PMC6176609 30224463

[5] Pho M, Berrada Y, Gunda A, et al. Nuclear shape is affected differentially by loss of lamin A, lamin C, or both lamin A and C. MicroPubl Biol. 2024;2024. doi: 10.17912/micropub.biology.001103 PMC10910297 38440331

[6] Kalukula Y, Stephens AD, Lammerding J, et al. Mechanics and functional consequences of nuclear deformations. Nat Rev Mol Cell Biol. 2022;23(9):583–602. doi: 10.1038/s41580-022-00480-z PMC9902167 35513718

[7] De Vos WH, Houben F, Kamps M, et al. Repetitive disruptions of the nuclear envelope invoke temporary loss of cellular compartmentalization in laminopathies. Hum Mol Genet. 2011;20(21):4175–4186. doi: 10.1093/hmg/ddr344 21831885

[8] Helfand BT, Wang Y, Pfleghaar K, et al. Chromosomal regions associated with prostate cancer risk localize to lamin B-deficient microdomains and exhibit reduced gene transcription. J Pathol. 2012;226(5):735–745. doi: 10.1002/path.3033 22025297

[9] Denais CM, Gilbert RM, Isermann P, et al. Nuclear envelope rupture and repair during cancer cell migration. Science. 2016;352(6283):353–358. doi: 10.1126/science.aad7297 PMC4833568 27013428

[10] Raab M, Gentili M, de Belly H, et al. ESCRT iii repairs nuclear envelope ruptures during cell migration to limit DNA damage and cell death. Science. 2016;352(6283):359–362. doi: 10.1126/science.aad7611 27013426

[11] Pfeifer CR, Xia Y, Zhu K, et al. Constricted migration increases DNA damage and independently represses cell cycle. Mol Biol Cell. 2018;29(16):1948–1962. doi: 10.1091/mbc.E18-02-0079 PMC6232975 29742017

[12] Cho S, Vashisth M, Abbas A, et al. Mechanosensing by the lamina protects against nuclear rupture, DNA damage, and cell-cycle arrest. Dev Cell. 2019;49(6):920–935.e5. doi: 10.1016/j.devcel.2019.04.020 PMC6581604 31105008

[13] Stephens AD, Liu PZ, Kandula V, et al. Physicochemical mechanotransduction alters nuclear shape and mechanics via heterochromatin formation. Mol Biol Cell. 2019;30(17):2320–2330. doi: 10.1091/mbc.E19-05-0286 PMC6743459 31365328

[14] Hatch EM, Hetzer MW. Nuclear envelope rupture is induced by actin-based nucleus confinement. J Cell Biol. 2016;215(1):27–36. doi: 10.1083/jcb.201603053 PMC5057282 27697922

[15] Berg IK, Currey ML, Gupta S, et al. Transcription inhibition suppresses nuclear blebbing and rupture independent of nuclear rigidity. J Cell Sci. 2023;136(20). doi: 10.1242/jcs.261547 PMC10660790 37756607

[16] Prince K, Lin K, Li A, et al. Transcriptional activity generates chromatin motion that drives nuclear blebbing. Mol Biol the Cell. Ahead Print. 2025 [cited 2026 September 16]; doi: 10.1091/mbc.E25-05-0237 PMC13729844 42747998

[17] Bunner S, Huang K, Shah A, et al. Changes in nuclear and actin mechanics from G1 to G2 affect nuclear integrity. J Cell Sci. 2026;139(12). doi: 10.1242/jcs.264118 PMC12989073 41549998

[18] Mouelhi M, Saffon A, Roinard M, et al. Mitosis sets nuclear homeostasis of cancer cells under confinement, eLife Life. 2024;13: RP94975. doi: 10.7554/eLife.94975.1

[19] Dahl KN, Engler AJ, Pajerowski JD, et al. Power-law rheology of isolated nuclei with deformation mapping of nuclear substructures. Biophys J. 2005;89(4):2855–2864. doi: 10.1529/biophysj.105.062554 PMC1366783 16055543

[20] Rowat AC, Lammerding J, Ipsen JH. mechanical properties of the cell nucleus and the effect of emerin deficiency. Biophys J. 2006;91(12):4649–4664. doi: 10.1529/biophysj.106.086454 PMC1779937 16997877

[21] Stephens AD, Banigan EJ, Adam SA, et al. Chromatin and lamin a determine two different mechanical response regimes of the cell nucleus. Mol Biol Cell. 2017;28(14):1984–1996. doi: 10.1091/mbc.e16-09-0653 PMC5541848 28057760

[22] Hobson CM, Kern M, O’brien ET, et al. Correlating nuclear morphology and external force with combined atomic force microscopy and light sheet imaging separates roles of chromatin and lamin A/C in nuclear mechanics. Mol Biol Cell. 2020;31(16):1788–1801. doi: 10.1091/mbc.E20-01-0073 PMC7521857 32267206

[23] Lele TP, Dickinson RB, Gundersen GG. mechanical principles of nuclear shaping and positioning. J Cell Biol. 2018;217(10):3330–3342. doi: 10.1083/jcb.201804052 PMC6168261 30194270

[24] Tocco VJ, Li Y, Christopher KG, et al. The nucleus is irreversibly shaped by motion of cell boundaries in cancer and non-cancer cells. J Cell Physiol. 2018;233(2):1446–1454. doi: 10.1002/jcp.26031 PMC5673577 28542912

[25] Le Berre M, Aubertin J, Piel M. Fine control of nuclear confinement identifies a threshold deformation leading to lamina rupture and induction of specific genes. Integr Biol (Camb). 2012;4(11):1406–1414. doi: 10.1039/c2ib20056b 23038068

[26] Tamiello C, Kamps MAF, van den Wijngaard A, et al. Soft substrates normalize nuclear morphology and prevent nuclear rupture in fibroblasts from a laminopathy patient with compound heterozygous LMNA mutations. Nucleus. 2013;4(1):61–73. doi: 10.4161/nucl.23388 PMC3585029 23324461

[27] Mistriotis P, Wisniewski EO, Bera K, et al. Confinement hinders motility by inducing RhoA-mediated nuclear influx, volume expansion, and blebbing. J Cell Biol. 2019;218(12):4093–4111. doi: 10.1083/jcb.201902057 PMC6891075 31690619

[28] Bunner S, Prince K, Pujadas Liwag EM, et al. Decreased DNA density is a better indicator of a nuclear bleb than lamin B loss. J Cell Sci. 2024;138(3). 10.1242/jcs.262082 PMC11883270 39501901

[29] Chu CG, Lang N, Walsh E, et al. Lamin B loss in nuclear blebs is rupture dependent whereas increased DNA damage is rupture independent. J Cell Sci. 2025;138(21). doi: 10.1242/jcs.263945 PMC12669966 41047936

[30] Clark M, Losada A, Jahng S, et al. Nuclear blebs are composed of variable chromatin states but consistently enrich transcription initiation relative to elongation. bioRxiv. 2026. doi: 10.1101/2026.03.10.710873 PMC13750403 42862039

[31] Currey ML, Kandula V, Biggs R, et al. A versatile micromanipulation apparatus for biophysical assays of the cell nucleus. Cell Mol Bioeng. New York, United States: Springer New York LLC; 2022. 10.1007/s12195-022-00734-y PMC9474788 36119136

[32] Young AM, Gunn AL, Hatch EM. BAF facilitates interphase nuclear membrane repair through recruitment of nuclear transmembrane proteins. Mol Biol Cell. 2020;31(15):1551–1560. doi: 10.1091/mbc.E20-01-0009 PMC7521799 32459568

[33] Shah P, Hobson CM, Cheng S, et al. Nuclear deformation causes DNA damage by increasing replication stress. Curr Biol. 2021;31(4):753–765.e6. doi: 10.1016/j.cub.2020.11.037 PMC7904640 33326770

[34] Pajerowski JD, Dahl KN, Zhong FL, et al. Physical plasticity of the nucleus in stem cell differentiation. Proc Natl Acad Sci USA. 2007;104(40):15619–15624. doi: 10.1073/pnas.0702576104 PMC2000408 17893336

[35] Davidson PM, Fedorchak GR, Mondésert-Deveraux S, et al. High-throughput microfluidic micropipette aspiration device to probe time-scale dependent nuclear mechanics in intact cells. Lab Chip. 2019;19(21):3652–3663. doi: 10.1039/C9LC00444K PMC6810812 31559980

[36] Golloshi R, Playter C, Freeman TF, et al. Constricted migration is associated with stable 3D genome structure differences in cancer cells. EMBO Rep. 2022;23(10):e52149. doi: 10.15252/embr.202052149 PMC9535800 35969179

[37] Pujadas Liwag EM, Acosta N, Almassalha LM, et al. Nuclear blebs are associated with destabilized chromatin-packing domains. J Cell Sci. 2025;138(3). doi: 10.1242/jcs.262161 PMC11883274 39878045

[38] Kono Y, Adam SA, Sato Y, et al. Nucleoplasmic lamin C rapidly accumulates at sites of nuclear envelope rupture with BAF and cGAS. J Cell Biol 221. 2022;221(12). doi: 10.1083/jcb.202201024 PMC9617480 36301259

[39] Robijns J, Molenberghs F, Sieprath T, et al. In silico synchronization reveals regulators of nuclear ruptures in lamin A/C deficient model cells. Sci Rep. 2016;6(1):30325. doi: 10.1038/srep30325 PMC4962089 27461848

[40] Borowski N, Li A, Stephens AD. Topoisomerase I inhibition suppresses nuclear blebbing via RNA pol ii stalling and nuclear stiffening. bioRxiv. 2026. doi: 10.1101/2026.01.09.698681

[41] Imbalzano KM, Cohet N, Wu Q, et al. Nuclear shape changes are induced by knockdown of the SWI/SNF ATPase BRG1 and are independent of cytoskeletal connections. PLOS ONE. 2013;8(2):e55628. doi: 10.1371/journal.pone.0055628 PMC3566038 23405182

[42] Barutcu AR, Lajoie BR, Fritz AJ, et al. SMARCA4 regulates gene expression and higher-order chromatin structure in proliferating mammary epithelial cells. Genome Res. 2016;26(9):1188–1201. doi: 10.1101/gr.201624.115 PMC5052043 27435934

[43] Patta I, Zand M, Lee L, et al. Nuclear morphology is shaped by loop-extrusion programs. Nature. 2024;627(8002):196–203. doi: 10.1038/s41586-024-07086-9 PMC11052650 38355805

[44] Shimi T, Pfleghaar K, Kojima S-I, et al. The A- and B-type nuclear lamin networks: microdomains involved in chromatin organization and transcription. Genes Dev. 2008;22(24):3409–3421. doi: 10.1101/gad.1735208 PMC2607069 19141474

[45] Stephens AD, Liu PZ, Banigan EJ, et al. Chromatin histone modifications and rigidity affect nuclear morphology independent of lamins. Mol Biol Cell. 2018;29(2):220–233. doi: 10.1091/mbc.E17-06-0410 PMC5909933 29142071

[46] Vahabikashi A, Sivagurunathan S, Nicdao FAS, et al. Nuclear lamin isoforms differentially contribute to LINC complex-dependent nucleocytoskeletal coupling and whole-cell mechanics. Proc Natl Acad Sci USA. 2022;119(17):e2121816119. doi: 10.1073/pnas.2121816119 PMC9170021 35439057

[47] Gurvich N, Tsygankova OM, Meinkoth JL, et al. Histone deacetylase is a target of valproic acid-mediated cellular differentiation. Cancer Res. 2004;64(3):1079–1086. doi: 10.1158/0008-5472.CAN-03-0799 14871841

