# Supplementary material for: Nuclear blebs are rupture‑prone plastic deformations
Source: Nucleus. 2026 Nov 4;17(1):2735710. doi: 10.1080/19491034.2026.2735710 (PMC13761373; doi:10.1080/19491034.2026.2735710)
Supplement: Supplementary file 1 [file KNCL_A_2735710_SM9938.pdf]

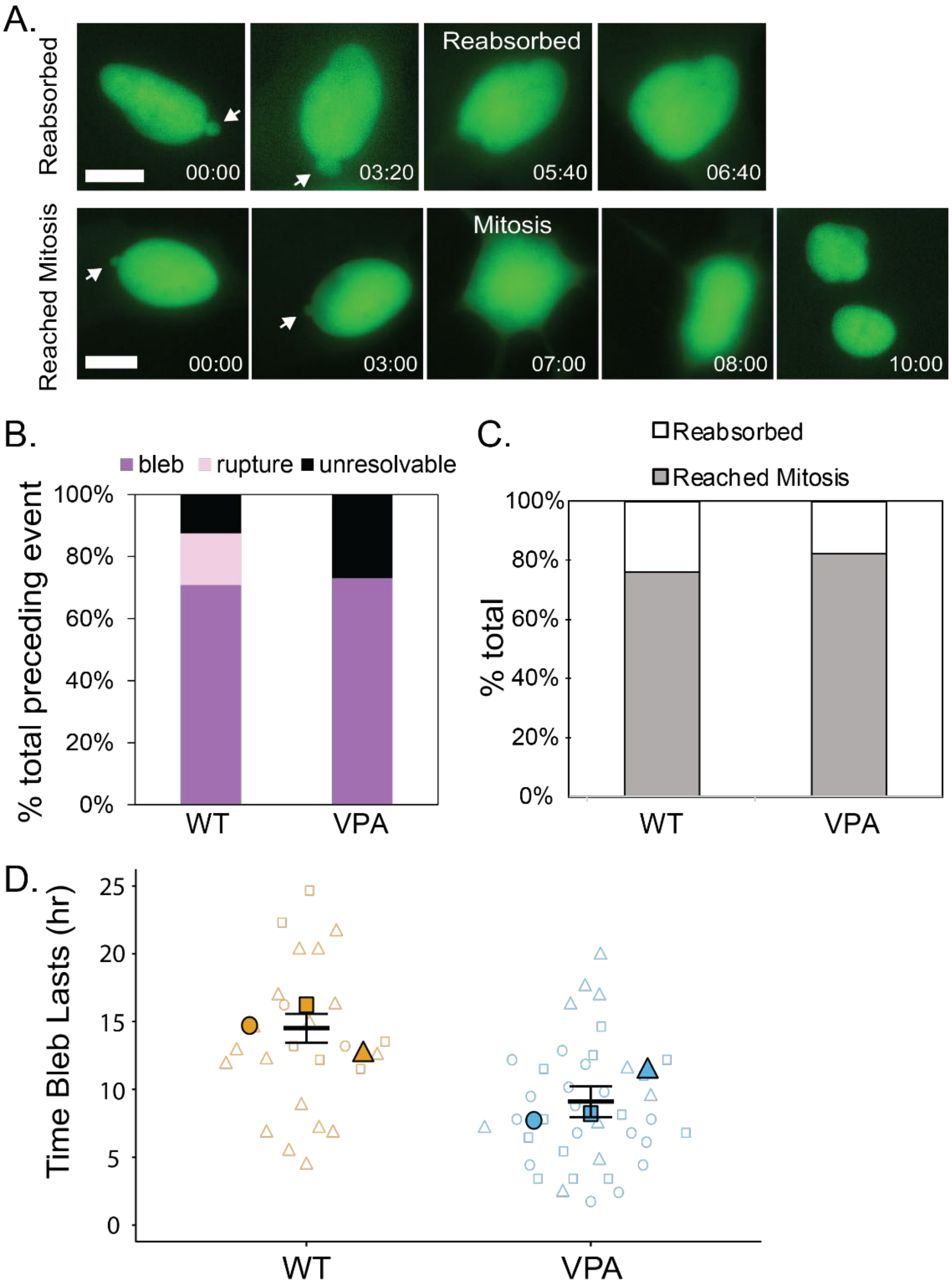

Supplemental Figure 1. Nuclear blebs predominantly persist till mitosis entry in HT1080 cells. (A) Example images of blebbed nuclei with NLS-GFP lasting till mitotic entry or upon reabsorption. White arrow indicates a nuclear bleb. (B) Graph of the % total preceding events for bleb formation vs. rupture and reporting unresolvable cases where they occur simultaneously. HT1080 WT n= 24 and VPA n = 26. (C) Graph of the total percentage of blebbed nuclear that reached mitosis vs. were reabsorbed. (D) Super plot of time that a bleb lasts in hours for wild type (WT) and VPA-treated nuclei (VPA). Biological triplicate with n > 25 nuclei with a bleb total. Error bars represent the standard error, and statistical tests are unpaired two-tailed Students' t-test; significance is denoted by * = p < 0.05, ** = p < 0.05, and *** = p < 0.05. Scale bar = 10µm.

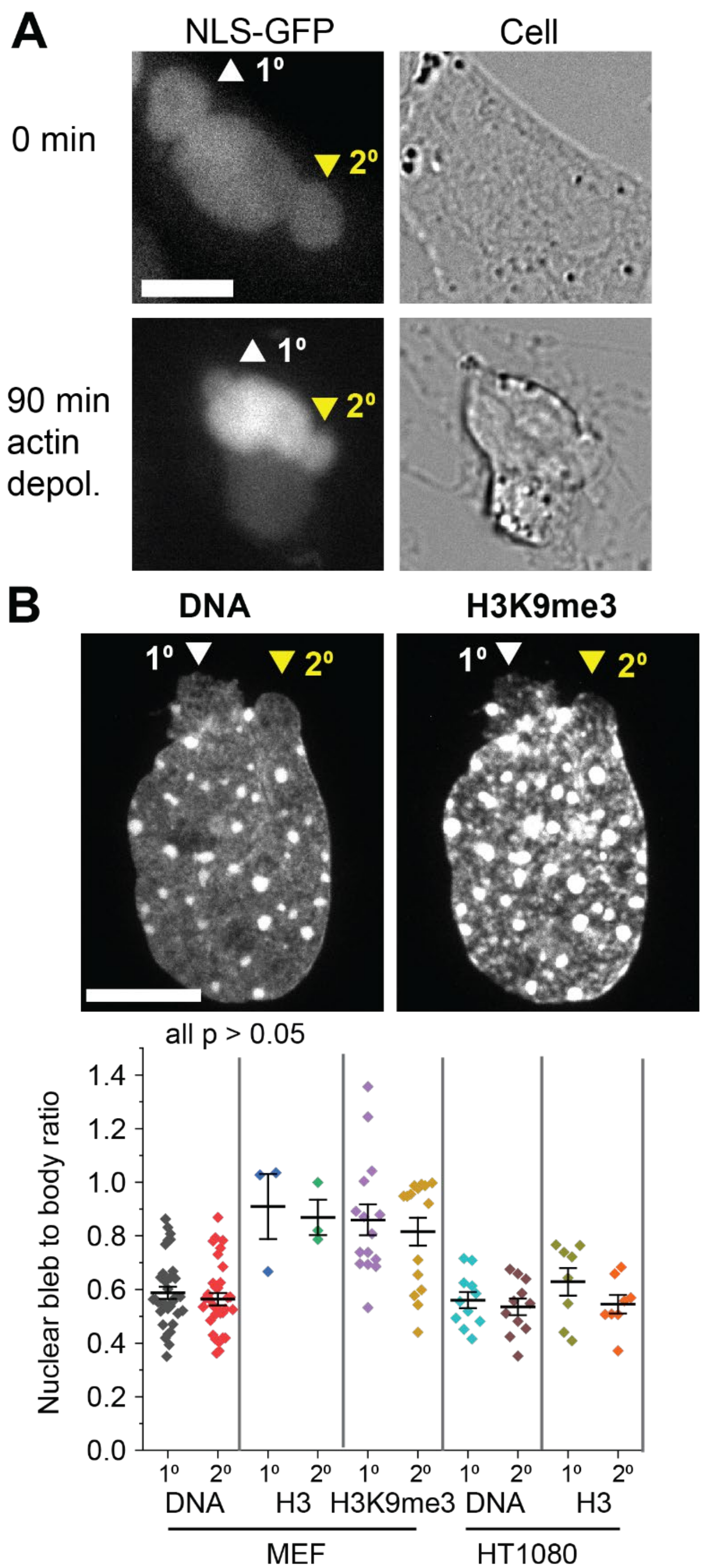

Supplemental Figure 1. Primary and secondary nuclear blebs display similar plasticity to actin depolymerization and chromatin content. (A) Example images of the nuclear bleb and body with NLS-GFP and the cell with transmitted light over the course of one and half hours after actin depolymerization with Latrunculin A (LatA) treatment. (B) Example images and graphs comparing the primary (10) and secondary nuclear blebs on the same nucleus. Example images are of DNA stain Hoechst 33342 and immunofluorescence constitutive heterochromatin marker H3K9me3 in MEFs. Graph compares chromatin content between the primary and secondary blebs in MEFs via DNA (n = 33), H3 (n =3), and H3K9me3 (n= 15) and in HT1080 via DNA (n = 11) and H3 (N=8).Error bars represent the standard error, and statistical tests are paired two-tailed Students' t-test; significance where all measurements were insignificant $p > 0.05$. Scale bar = 10μm.
